# Correction to: Implantable brain–computer interface for neuroprosthetic-enabled volitional hand grasp restoration in spinal cord injury

**DOI:** 10.1093/braincomms/fcac143

**Published:** 2022-06-23

This is a correction to: Iahn Cajigas, Kevin C. Davis, Benyamin Meschede-Krasa, Noeline W. Prins, Sebastian Gallo, Jasim Ahmad Naeem, Anne Palermo, Audrey Wilson, Santiago Guerra, Brandon A. Parks, Lauren Zimmerman, Katie Gant, Allan D. Levi, W. Dalton Dietrich, Letitia Fisher, Steven Vanni, John Michael Tauber, Indie C. Garwood, John H. Abel, Emery N. Brown, Michael E. Ivan, Abhishek Prasad, Jonathan Jagid, Implantable brain–computer interface for neuroprosthetic-enabled volitional hand grasp restoration in spinal cord injury, *Brain Communications*, Volume 3, Issue 4, 2021, fcab248, https://doi.org/10.1093/braincomms/fcab248

In the originally published version of this manuscript, the following competing interest was omitted in error.

Dr. Brown is a cofounder of PASCALL, a company developing closed loop physiological control systems for anesthesiology.

This error has been corrected.

